# Effects of Cations on the Behaviour of Lipid Cubic Phases

**DOI:** 10.1038/s41598-017-08438-4

**Published:** 2017-08-15

**Authors:** Christopher Brasnett, Georgia Longstaff, Laura Compton, Annela Seddon

**Affiliations:** 1 H.H. Wills Physics Laboratory, Tyndall Avenue, University of Bristol, Bristol, BS8 1FD UK; 2 Bristol Centre for Functional Nanomaterials, HH Wills Physics Laboratory, Tyndall Avenue, University of Bristol, Bristol, BS8 1FD UK

## Abstract

Inverse bicontinuous cubic structures formed by lipids have been demonstrated in a wide variety of applications, from a host matrix for proteins for crystallisation, to templates for nanoscale structures. Recent work has focused on tuning their properties to realize such applications, often by manipulating the structure by introducing other lipids with different properties such as charge or packing. However, they are often prepared in the presence of solutions containing salt, counteracting the effects, for example, charged lipids, and fundamentally changing the structures obtained. Here, we demonstrate the delicate interplay between electrostatic swelling in bicontinuous structures formed by monoolein (MO) doped with both negatively charged dioleyl phosphatidylglycerol (DOPG), and zwitterionic dioleyl phosphatidylethanolamine (DOPE), with the addition of mono- and divalent salts. The effect of adding salt to the charged phase changes the structure from the primitive cubic (${{\bf{Q}}}_{II}^{P}$) to the double diamond phase (${{\bf{Q}}}_{II}^{D}$) whilst still allowing for modest increases in lattice parameter of up to a nanometer. Contrasting this, the addition of salts to the non-charged phase, has minimal effect on the lattice parameter but now the transition from the (${{\bf{Q}}}_{II}^{D}$) to the inverse hexagonal phase (H_*II*_) is observed occurring at higher mole fractions of DOPE than in pure water.

## Introduction

The self-assembly of biological amphiphiles such as lipids continues to be a rich area of research, from the fundamental understanding of the formation of biological membranes, through technological applications such as membrane protein crystallization and templating of nanoscale structures^1–5^. This is driven in part driven through the diverse range of structures which they are able to form - structures which are based on the desire for the lipids to adopt phases with varying degrees of negative curvature^6^. These can be simple fluid lamellar phases, such as those found in the plasma membrane of cells; however more complex inverse bicontinuous cubic phases are also seen, in model systems, and in some biological systems such as those reported by Almsherqi^7^ and Staudegger^8^, as well as being thought to be the intermediate stages of membrane fusion^9^. However much of the work undertaken in illuminating the phase behaviour of lipids is done so in water, which does not reflect the complex mixture of salts and small molecules either within the cell, the model membrane systems used for protein folding experiments, or the conditions encountered during protein crystallization, despite extensive evidence that salt concentrations have an effect on biological function^10, 11^.

Since its potential as a host for membrane protein crystallization was first demonstrated in 1996^12^, work on understanding the inverse bicontinuous cubic phases and how they may be tuned to allow more successful protein crystallisation has shown that *in cubo* crystallisation is at present likely to continue to be the method of choice for growing membrane protein crystals^13, 14^. However it is clear when one considers that unique membrane protein structures in the protein data bank still only number 686^15^, compared to up to 40,000 soluble proteins^16^, that there is still much to be learned. One issue that still exists is in the understanding of the effects of the interactions between the proteins, the lipids and the various crystallisation precipitants and buffers that are required for a successful crystallisation trial. The second issue is that of the natural requirement for certain proteins - such as the potassium channel KcsA^17^ - to be bound to particular lipids for function.

**Figure 1:**
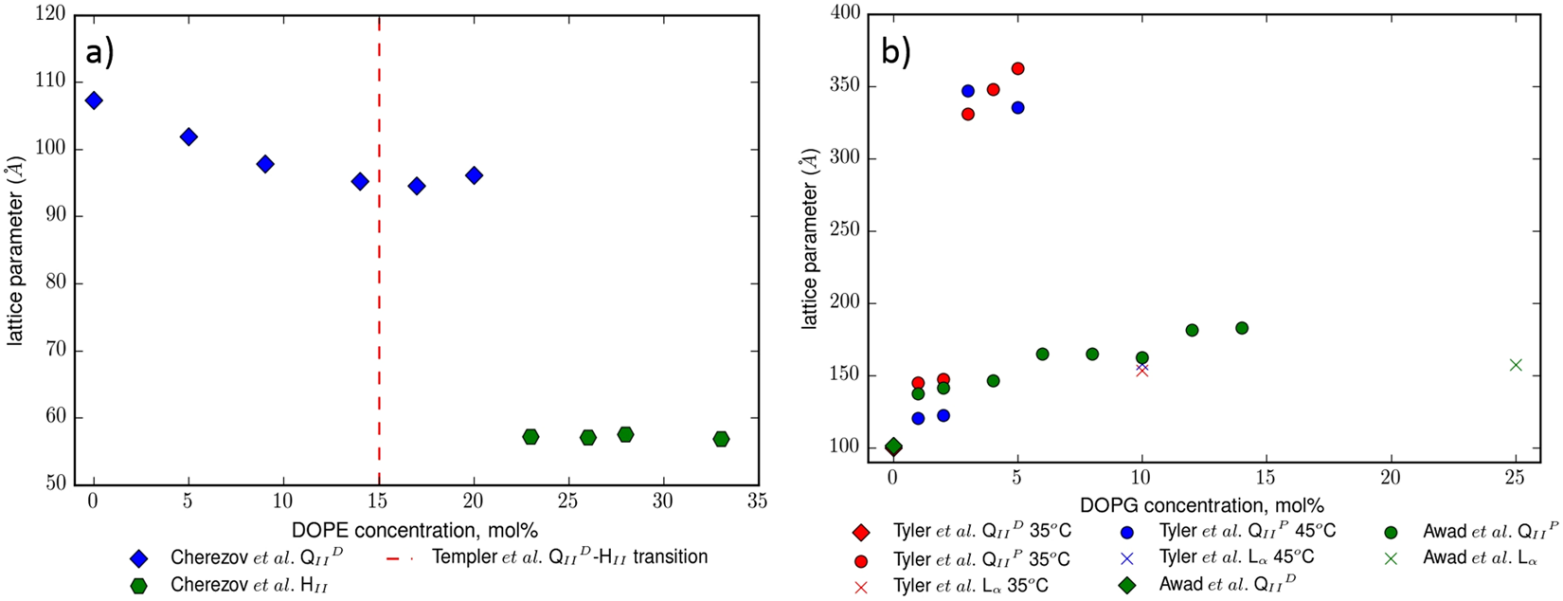
An overview of the present literature understanding of the phase behaviour of Monoolein when doped with increasing molar proportions of (**a**) DOPE and (**b**) DOPG. (**a**) demonstrates that upon addition of zwitterionic DOPE, Cherezov *et al*.^19^ found a slight decrease in the ${{\rm{Q}}}_{II}^{D}$ phase lattice parameter, before the system took on the inverse hexagonal phase at above 20 mol%, a similar finding to phase work (with no lattice parameter information) done by Templer *et al*.^20^. (**b**) Shows that the work of Tyler *et al*.^21^ and Awad *et al*.^25^ finds an initial phase change from ${{\rm{Q}}}_{II}^{D}$ to ${{\rm{Q}}}_{II}^{P}$ upon addition of the anionic lipid DOPG, a subsequent swelling of the ${{\rm{Q}}}_{II}^{P}$ phase, before a further phase change to the L_*α*_. We note, however, that Tyler *et al*. undertook their experiments at temperatures too high for practical application purposes, and that Awad *et al*. undertook their measurements in a 10 mM PIPES buffer.

Previous work by Conn^18^, Cherezov^19^, and co-workers have focused particularly on the effects of common precipitants and additives to monoolein (MO), the lipid most often used for membrane protein crystallisation *in cubo*. The 2002 study by Cherezov *et al*. described lipid tailoring of the cubic phase formed by MO by the addition of a number of commonly encountered lipids. As we show in Fig. 1a (along with a related study by Templer and co-workers^20^), it was demonstrated that MO can tolerate up to 20 mole % dioleyl phosphatidylethanolamine (DOPE) before the onset of the formation of the highly negatively curved Inverse Hexagonal (H_*II*_) phase, and addition of DOPE caused a contraction in the lattice parameter of 1 nm when compared to pure MO.

More recently, and as we show in Fig. 1b, work by Tyler *et al*.^21^ has shown that careful selection of anionic and zwitterionic additive lipids to MO allows tuning of the size of the lattice parameter of the assembled phase to produce arguably the largest lipid cubic phases to date. The aforementioned work of Cherezov *et al*.^19^ had also previously probed the addition of the anionic phospholipid dioleylphosphatidyl serine (DOPS) showing that the Primitive cubic (${{\rm{Q}}}_{II}^{P}$) phase is formed at low molar proportitons of inclusion of DOPS, before a largely swollen Double Diamond cubic (${{\rm{Q}}}_{II}^{D}$) phase was seen as the proportion of DOPS was increased^19, 22^. The effect of anionic lipid headgroups on monoolein cubic phases was first noted by Engblom *et al*.^23^, who found that incorporation of small quantities of distearoylphosphatidylglycerol (DSPG) can induce and swell the ${{\rm{Q}}}_{II}^{P}$ phase. The distearoyl tails of DSPG are saturated, however, and so in room-temperature conditions (as studied by Cherezov *et al*.) will have an increased elastic moduli^24^, and be less fluid, so are not presented for comparison in Fig. 1b. Similar swelling effects for the cubic gyroid (${{\rm{Q}}}_{II}^{P}$) phase have been observed upon the addition of the cationic lipid dioleoyltrimethylammoniumpropane (DOTAP) to MO^5^.

Although the results of Tyler *et al*. are impressive, it should be noted, (as indeed it was by the authors^21^) that the addition of salts to structures formed from charged lipids such as dioleyl phosphatidylglycerol (DOPG) may well affect the nature of the structures formed. Furthermore, their experiments were undertaken at elevated temperatures of 35 °C and 45 °C, temperatures which would be unfavourable for the production of protein crystals. That the presence of salt has an effect has been hinted at previously by Awad *et al*.^25^, who demonstrated that for buffer suspensions of large unilamellar vesicles (LUVs) consisting of MO and DOPG, the most stable phase could be changed from L_*α*_ to cubic through addition of Ca^2+^ ions, which we show again in Fig. 1b. Although the authors noted that the change in phase stability was brought about through an increase of the spontaneous curvature of the membrane, work undertaken since has additionally demonstrated that the presence of buffers in model lipid systems can reduce the Gaussian curvature elastic energy stored in the membrane, resulting in an interplay of phenomena affecting the phase stability^26^. Furthermore, it has been noted^18, 27^ that the effect of salt ions on biological systems can be defined in terms of their position in the Hofmeister series. In the Hofmeister series, anions and cations can be classed on a spectrum of chaotropes and kosmotropes, varying from water structure-breakers and water structure makers respectively. Regarding cations, the ordering of ions relevant to this work can be given as Cs^+^ < Rb^+^ < ${{\rm{NH}}}_{4}^{+}$ < K^+^ < Na^+^ < Li^+^ < Ca^2+^ < Mg^2+^ < Zn^2+^ in increasing kosmotropic strength^28^. Anions are ordered SCN^−^ < ClO^4−^ < Br^−^ < NO^3−^ < Cl^−^ < SO^4−^, similarly for increasing kosmotropic strength^29^, with the Chloride ion lying on the boundary between being a kosmotrope and a chaotrope. In the context of lipid membranes, the structure making ability of kosmotropes promotes the existence of more highly-curved phases (Ie. towards the H_*II*_ phase on the Gaussian curvature spectrum), due to their being excluded from the interfacial region of the membrane-solution system^18, 30^.

Whilst it is clear that much effort has been applied to understanding the effect of lipid tailoring and the effects of complex crystallization screens, there is a still a gap in the literature for a systematic understanding of tailoring the lipid composition of MO cubic phases in the presence of simple salt solutions. Furthermore, whilst the effect of anions on membranes appears to be greater than that of cations^31^, precise cation effects have not previously been investigated. Therefore in this work, we systematically investigate the effects of mono- and divalent salts on the structural parameters of a lipid cubic phase formed from a mixture of the monoacyl glycerol lipid, monoolein (MO) in the presence of either the anionic phospholipid dioleylphosphotidyl glycerol (DOPG) or the zwitterionic phospholipid dioleylphosphotidyl ethanolamine (DOPE) in order to better understand the interplay between the electrostatic and packing behaviour of lipids, and how the parameters governing the formation of lipid cubic phases may be tuned.

## Methods and Materials

Monoolein (MO) was received as gift from Danisco and used without further preparation. DOPG and DOPE were obtained from Avanti Polar Lipids. Dicholoromethane (DCM), NaCl, LiCl and CaCl_2_ were obtained from Sigma Aldrich. MilliQ water was used in the preparation of all samples. To prepare Monoolein doped with DOPG or DOPE, lipids were dissolved separately in (DCM), to yield 0.1 M solutions. Samples of varying molar ratios of MO and DOPG, or DOPE were prepared by mixing these solutions in the required ratio. The mixed lipids in DCM were then pipetted directly into borosilicate glass X-ray capillaries (Gulmay Medical). The capillaries were then left for approximately 36 hours in order for the majority of the DCM to evaporate. After this time, the remaining solution was dispersed using a stream of nitrogen gas to create a lipid film on the inside of the capillary. Samples were then hydrated with 50 *μ*L of water, or salt solution of the desired concentration, followed by centrifugation of the capillary at 1600 rpm for 1 minute, and sealing with UV-curable adhesive (Norland Optical Adhesive) for 30 minutes. Samples were equilibrated for a minimum of 5 days before scattering patterns were taken. X-ray scattering was performed on a SAXSLAB Ganesha 300XL instrument in a q range of 0.015–0.65 Å^−1^, with an exposure time of 600 seconds per sample.

### Data Availability

The datasets generated during and/or analysed during the current study are presented within the published manuscript. All raw data will be made freely available by the corresponding author upon reasonable request.

## Results and Discussion

**Figure 2:**
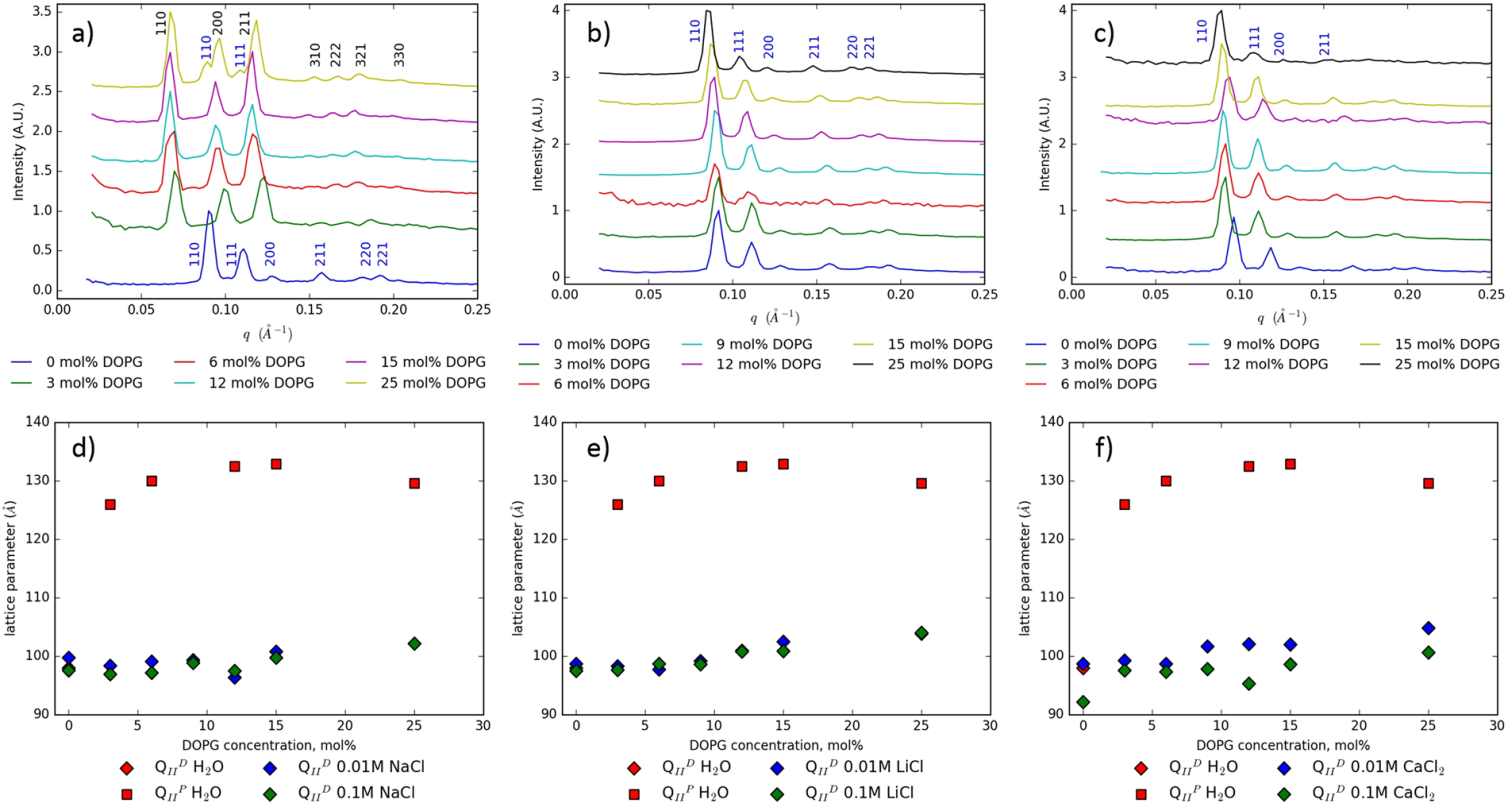
The effect of changing the hydrating salt cation in lipid systems of MO and DOPG. Bragg peaks in figs (**a–c**) have been indexed according to the Miller indicies of the reflection place, with Blue labels indicating a ${{\rm{Q}}}_{II}^{D}$ plane, and Black ${{\rm{Q}}}_{II}^{P}$ one. (**a**) 1D radially integrated SAXS pattern of increasing DOPG concentration measured in water; (**b**) increasing DOPG concentration measured in 0.1 M LiCl; (**c**) increasing DOPG concentration measured in 0.1 M CaCl_2_. The effect of two concentrations - compared to water - of (**d**) NaCl on the phase and lattice parameter of a MO:DOPG cubic phase; (**e**) LiCl on the phase and lattice parameter of a MO:DOPG cubic phase; (**f**) CaCl_2_ on the phase and lattice parameter of a MO: DOPG cubic phase. Further scattering 1D SAXS patterns of the NaCl and LiCl systems are in the Supplementary Information, Figures S1–3.

Figure 2a shows the 1D scattering patterns obtained from MO with increasing amounts of DOPG hydrated in water. In excess water, MO alone forms a ${{\rm{Q}}}_{II}^{D}$ phase with a lattice parameter of around 100 Å^−1^ ^32^. The addition of DOPG to MO causes the cubic phase to convert from a ${{\rm{Q}}}_{II}^{D}$ to a ${{\rm{Q}}}_{II}^{P}$ phase, with the lattice parameter increasing from 98 Å in the ${{\rm{Q}}}_{II}^{D}$ phase to around 135 Å in the ${{\rm{Q}}}_{II}^{P}$ phase^19, 21^. This has been explained by the build-up of membrane charge density, leading to increased electrostatic repulsion between charged headgroups. In turn, this leads to an increase in the headgroup area, which decreases the packing parameter and thus decreases the Gaussian curvature, leading to a flatter ${{\rm{Q}}}_{II}^{P}$ phase, a mechanism that has been observed previously for both charged and neutral lipids^19, 33–37^. The increase in lattice parameter arises from the increase in membrane charge density and electrostatic repulsion which again increases the effective headgroup area, and causes a decrease in the spontaneous monolayer curvature and as such a larger lattice parameter. At the highest proportion of anionic lipid examined (25%), the 1D integrated SAXS profile in Fig. 2a exhibits extra peaks, arising from a coexisting ${{\rm{Q}}}_{II}^{D}$ phase of lattice parameter 100 Å. Such coexistance is in agreement with previous studies^19^, driven by the interplay between the demands of a now lack of headgroup hydration and the increase in area due to charge.

### Salt effects on electrostatic lipid systems

#### System size is chaotropically dependent

Although we observe the expected phase change from ${{\rm{Q}}}_{II}^{D}$ to ${{\rm{Q}}}_{II}^{P}$ upon the addition of the anionic lipid DOPG in water, in the presence of small concentrations (0.01 M) of salt solution, the same lipid systems will revert back to the ${{\rm{Q}}}_{II}^{P}$ phase. This is evidenced by the 1D scattering patterns in Fig. 2b and c of lipid systems comprising of MO and increasing proportions of DOPG in the presence of identical concentrations (0.1 M) of LiCl and CaCl_2_ respectively. Further scattering patterns for the rest of Fig. 2 are in the Supplementary Information, Figures S1–3. The increased membrane charge density responsible for the formation of the ${{\rm{Q}}}_{II}^{P}$ is now screened by the presence of salt, reducing the effective headgroup area and leading to the formation of the more curved ${{\rm{Q}}}_{II}^{D}$ phase. In the presence of monovalent salts, as the mole fraction of DOPG increases, slight swelling, as shown in Fig. 2d and e, of the ${{\rm{Q}}}_{II}^{D}$ phase is observed due to incomplete screening of the increasing DOPG charge. The swelling is approximately 5 Å in NaCl, and 7 Å in LiCl. This swelling of neutral membranes by monovalent salts is known to occur via weakening of the van der Waals force through screening of charge fluctuations^26, 38^. Therefore, as the membrane charge fluctuations are reduced, the van der Waals force will overall become increasingly repulsive, resulting in the swelling observed. In addition to the monovalent Sodium and Lithium ions, the divalent Calcium ion was chosen as it is known to bind strongly to the DOPG headgroup, drastically reducing the headgroup area and leading to the formation of highly curved phases^24^. Addition of Ca^2+^ to DOPG shows a similar trend to the monovalent salts above, with the ${{\rm{Q}}}_{II}^{D}$ phases swelling by 10–12 Å, as shown in Fig. 2f.

Comparing the swelling of the ${{\rm{Q}}}_{II}^{D}$ phase in the presence of all three salts as compared to water, (as shown in Fig. 2d–f), it becomes evident that this follows the Hofmeister series. The effect of cations on protein stability according to the Hofmeister series is well understood^39^ and its effect on lipid bilayer stability though not as well developed is beginning to be appreciated^18, 30^. As discussed above, kosmotropes - such as the Ca^2+^ ion - stabilise the structure of bulk water, and so will not be found in the interfacial region of the water channels of the cubic phases^18, 40^. Therefore, as the head group area of membrane lipids is decreased, the lattice parameter of the ${{\rm{Q}}}_{II}^{D}$ phase correspondingly does so^41^. Considering the cations used in this work, Ca^2+^ is the strongest kosmotrope, followed by Li^+^ and finally Na^+^ which lies on the border between chaotrope and kosmotrope, explaining the observed increasing differences in swelling in Fig. 2.

#### Effect of salt concentration

**Figure 3:**
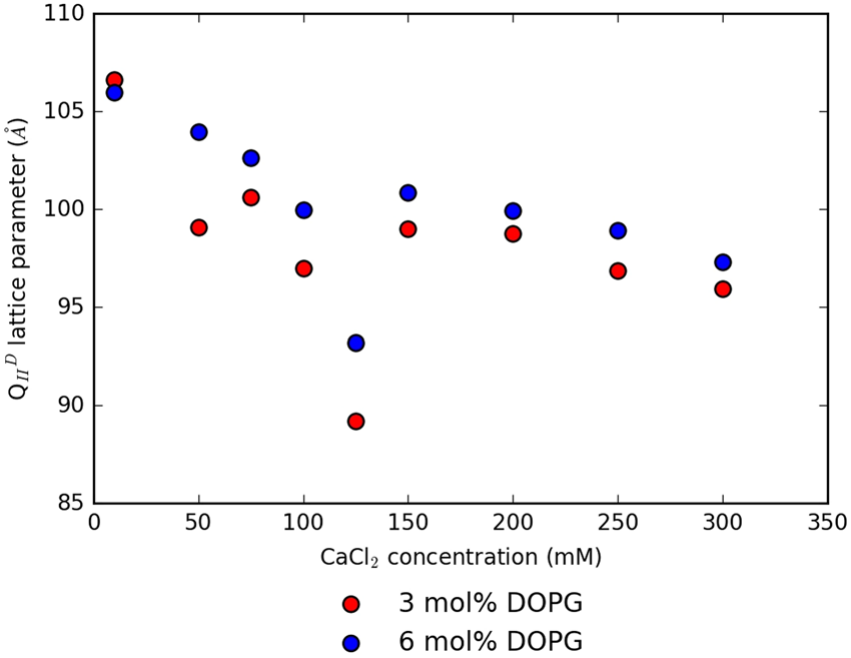
The effect of increasing the concentration of CaCl_2_ used to hydrate the monoolein/DOPG system on the ${{\rm{Q}}}_{II}^{D}$ lattice parameter. 1D SAXS patterns from which the data were evaluated are in the Supplementary Information, Figure S4.

In addition to the dependency of the phase behaviour of the system on salt content, we have additionally shown that the change in lattice parameter is dependent on the concentration of salt ions. As we show in Fig. 3, for systems of constant proportions of DOPG, the lattice parameter of the ${{\rm{Q}}}_{II}^{D}$ phase can vary up to 15 Å as the concentration of CaCl_2_ is increased.

Figure 3 demonstrates that an initial increase in the concentration of Ca^2+^ ions results in a marked decrease of the swelling observed in the ${{\rm{Q}}}_{II}^{D}$ phase. However, swelling is observed once more when the concentration of ions is increased further. The variation is a result of the delicate interplay between repulsive forces from charged membranes and the effects of the ions present in the system. The initial increase in Ca^2+^ ions screens the membrane charge and consequently reduces the swelling. We anticipate that once the membrane charge is completely screened, the addition of more Ca^2+^ ions to the system causes the system to swell again. The further decrease of the lattice parameter in the re-swelled system is characteristic of the system tending towards the inverse hexagonal phase^42^. The balance between the sources of electrostatic forces in the system are indicative that salt solution concentration in such systems has a subtle effect, and that single-concentration crystallisation screening experiments may not fully consider experimental optimisation^18, 43^.

### Salt effects on zwitterionic cubic lipid systems

**Figure 4:**
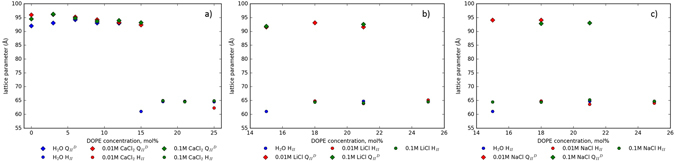
The effect of changing the hydrating salt cation in lipid systems of MO and DOPE. The effect of two concentrations - compared to water - of (**a**) CaCl_2_ on the phase and lattice parameter of a MO:DOPE cubic phase; (**b**) LiCl on the phase and lattice parameter of a MO:DOPE cubic phase; (**c**) NaCl on the phase and lattice parameter of a MO: DOPE cubic phase. In the presence of Ca^2+^ ions, ${{\rm{Q}}}_{II}^{D}$ phases of monoolein and DOPE show little variation in lattice parameter before a phase transition to H_*II*_. Upon hydration even with monovalent salt solutions, a complete ${{\rm{Q}}}_{II}^{D}$ to H_*II*_ phase transition is put off until the dominant force in the system is the membrane Gaussian curvature, with the system demonstrating phase coexistence at 18 mol% and 21 mol%. 1D SAXS patterns for the data are in the Supplementary Information, Figures S5–8.

In order to decouple the structural effects of the change in behaviour of DOPG in the presence of salt and the electrostatic effects we repeated the above salt concentration experiments but using DOPE in place of DOPG as there should be little to no driving force for DOPE to have any preferential reason to interact with CaCl_2_^24^ in the same manner as the anionic DOPG. As seen in Fig. 4a, in water, a ${{\rm{Q}}}_{II}^{D}$ phase with a lattice parameter comparable to MO alone is formed until a molar proportion of DOPE of 15% is reached, at which point there is a sharp transition to the inverse hexagonal phase, formed due to the increase in Gaussian curvature brought about from the addition of the highly type *II* DOPE. Addition of increasing concentrations of Ca^2+^ shows no appreciable change in the lattice parameter; the electrostatic swelling behaviour observed in the DOPG doped system is now not present. Whereas the addition of Ca^2+^ does delay the onset of the H_*II*_ phase, in water a transition to H_*II*_ was seen at 15% DOPE. In all concentrations of Ca^2+^, 15% DOPE remains in a ${{\rm{Q}}}_{II}^{D}$ phase and only becomes H_*II*_ at 18% PE, therefore suggesting that the presence of salt does indeed have an effect on the forces present in the system.

To further demonstrate this point, we have demonstrated in Fig. 4b and c that the phase transition to the H_*II*_ phase also follows a Hofmeister kosmotrope series. Figure 4b and c show that the MO/DOPE lipid system undergoes the ${{\rm{Q}}}_{II}^{D}$-H_*II*_ transition at higher molar proportions of DOPE than compared to the same system in water (Fig. 4a). The presence of kosmotropes is known to increase the Gaussian curvature propensity of the system^18, 40^, meaning the system may be expected to undergo the phase transition at lower molar proportions of DOPE. However, the shift in the transition point, and the specific ionic effects can be rationalised by considering the energetic cost of molecular reorganisation through the course of the transition. At low monolayer surface pressures, it is known that the presence of Ca^2+^ - but not Na^+^ - ions will is known to induce order in phospholipid tails through interaction with the phosphate moiety, forming domains^44^. As has been pointed out for the L_α_-H_*II*_ transition, the energetic barrier for the transition is high due to the differing topologies of the transition^45^. We demonstrate here that in the presence of salt, the point of transition to the H_*II*_ phase is increased as the molar proportion of DOPE is correspondingly increased, specifically in cations at the kosmotropic end of the Hofmeister series. This is due to the increased energetic cost of molecular reorganisation due to the formation of domains of DOPE in the ${{\rm{Q}}}_{II}^{D}$ phase in the presence of salt.

Further to membrane-binding effects, the Sodium ion lies on the boundary between being a kosmotrope and a chaotrope^46^, and so demonstrates a fluctuation in the position of the phase boundary. However, it is clear that as the cation of the salt used is changed from Na^+^ to Li^+^ to Ca^2+^, the presence of salt in the system delays the change in phase from ${{\rm{Q}}}_{II}^{D}$ to H_*II*_, a consequence of membrane swelling. Moreover, the weaker kosmotropic properties of the monovalent ions demonstrate a wide degree of phase coexistence between ${{\rm{Q}}}_{II}^{D}$ and H_*II*_, whereas the stronger Ca^2+^ ion does not. Whilst it is well known that the effect of kosmotropic anions is more significant than that of cations, this work has demonstrated that there is still a marked difference in the effect that the cations can produce^27, 47^.

## Conclusions

In this work, we have demonstrated that whilst doping lipid systems may enhance the achievable physical parameters, the presence of salt can severely affect both the phase and size of the resultant system. By varying both salt valency and concentration, we have shown that the combined effect the two have on lipid systems is non-negligible. In anionic systems, a phase change is observed from ${{\rm{Q}}}_{II}^{D}$ to ${{\rm{Q}}}_{II}^{D}$ upon the addition of any concentration of salt. The lattice parameter of the ${{\rm{Q}}}_{II}^{D}$ phase is subsequently dependent on the concentration of the salt used: a demonstration of the delicate interplay between the lipid packing in the system and the salt used to hydrate it. Additionally, we have demonstrated that similar effects, caused by the presence of salt ions, are present even in zwitterionic systems. The Gaussian curvature-driven ${{\rm{Q}}}_{II}^{D}$-H_*II*_ phase transition in zwitterionic systems is shown to be deterred by the presence of salt. Importantly, we have shown that for applications of cubic lipid systems, the choice of hydrating solution, even regarding the cation, is crucial for optimisation of system parameters. Such delicacy demonstrates the importance of multi-component screening prior to application. Ongoing studies in our group will demonstrate how the introduction of buffers may further affect membrane organisation in the presence of salt, providing an additional tool for tuning the size and packing of lipid bilayers. Furthermore, the work presented here, and our future work will inform the better design of model lipid systems for interactions with proteins, and potentially lead to an improvement in our understanding of membrane protein - lipid interactions.

## Electronic supplementary material

Supplementary Information
