# Supplementary material for: Effects of Cations on the Behaviour of Lipid Cubic Phases
Source: Sci Rep. 2017 Aug 15;7:8229. doi: 10.1038/s41598-017-08438-4 (PMC5557815; doi:10.1038/s41598-017-08438-4)
Supplement: Supplementary file 1 — Supplementary Information [file 41598_2017_8438_MOESM1_ESM.pdf]

Effects of Cations on the Behaviour of Lipid Cubic Phases

Christopher Brasnett[1], Georgia Longstaff[1], Laura Compton[1], Annela Seddon[1,2,*]

[1] HH Wills Physics Laboratory, Tyndall Avenue, University of Bristol, Bristol BS8 1FD

[2] Bristol Centre for Functional Nanomaterials, HH Wills Physics Laboratory, Tyndall Avenue, University of Bristol, Bristol BS8 1FD

[*] annela.seddon@bristol.ac.uk

Scattering Patterns

Anionic lipid systems

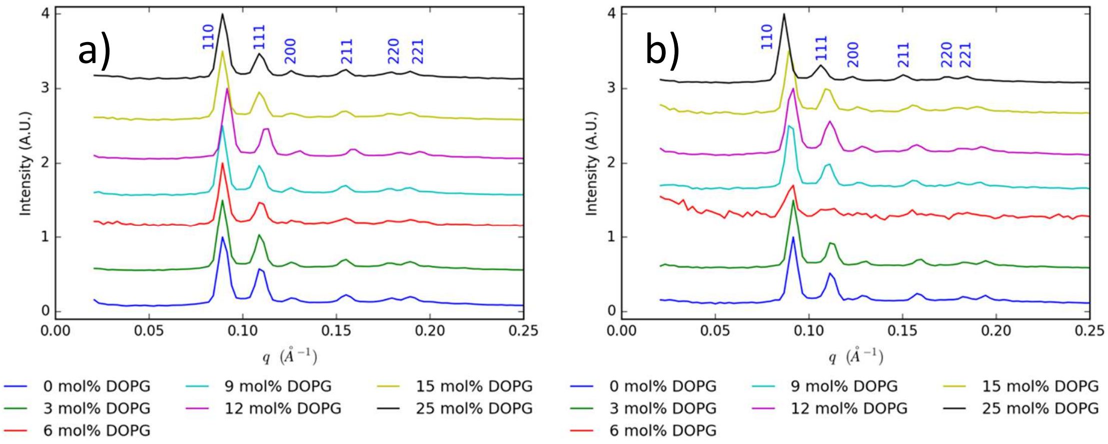

S1. 1D radially integrated scattering patterns of lipid systems of MO and DOPG in two concentrations of NaCl. (a) 0.01M NaCl; (b) 0.1M NaCl. Miller plane indicies of the Q_{II}^{D} phase are indicated in blue.

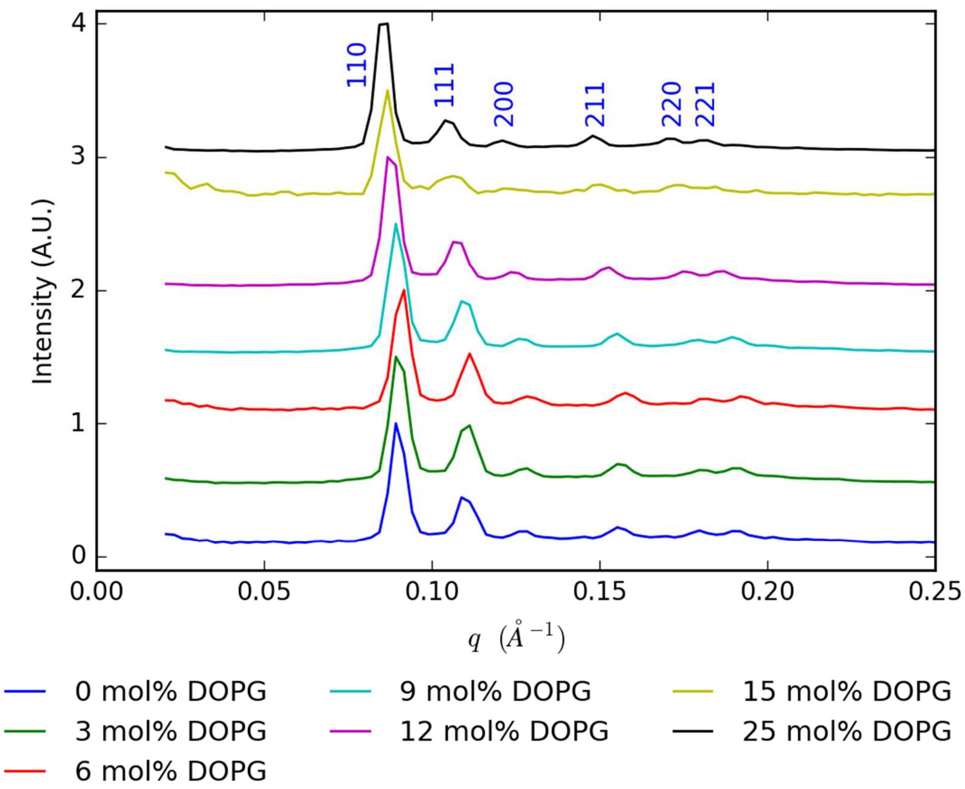

S2. 1D radially integrated scattering patterns of lipid systems of MO and DOPG in the presence of 0.01M LiCl. Miller plane indicies of the Q_{II}^{D} phase are indicated in blue.

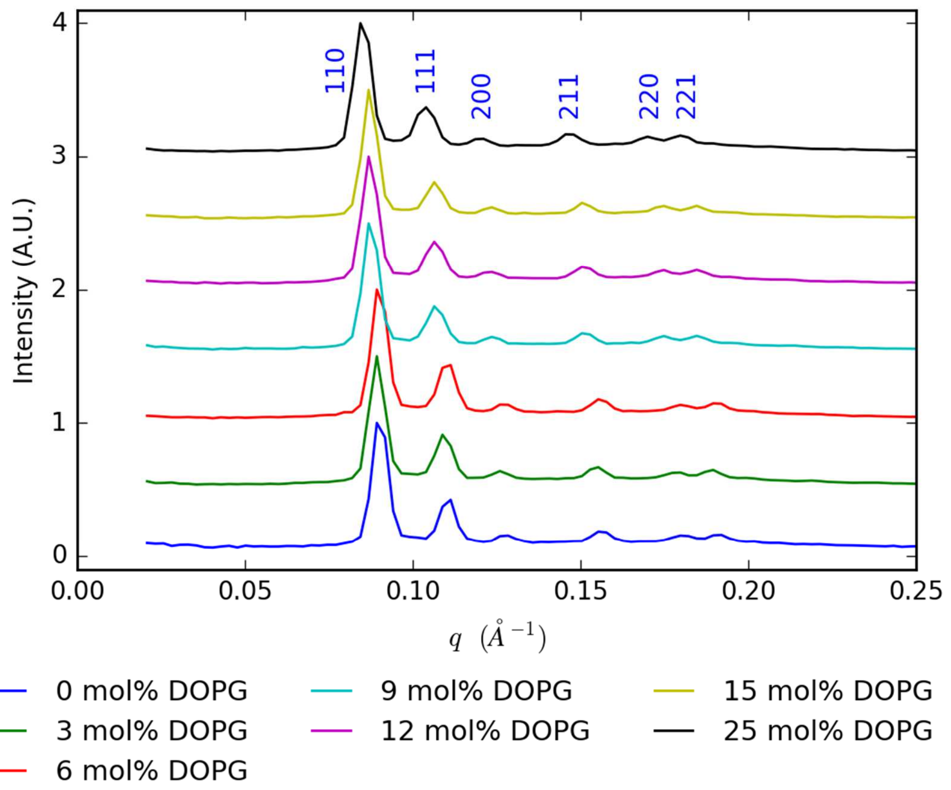

S3. 1D radially integrated scattering patterns of lipid systems of MO and DOPG in the presence of 0.01M $CaCl_2$. Miller plane indicies of the Q_{II}^{D} phase are indicated in blue.

Concentration

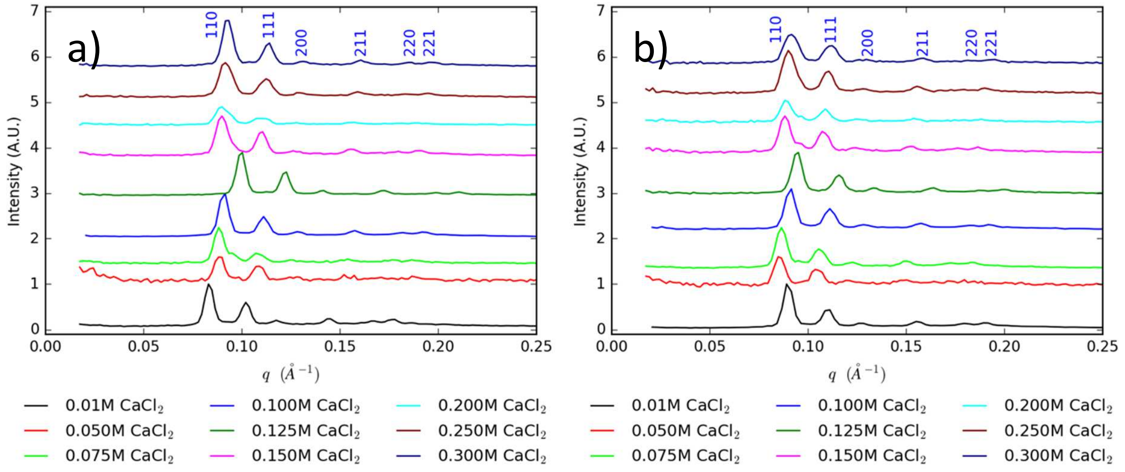

S4. 1D radially integrated scattering patterns of lipid systems of MO and DOPG, where the proportion of DOPG in the system is held constant, and the hydrating salt concentration is varied. (a) 3 mol% DOPG; (b) 6 mol% DOPG. Miller plane indicies of the Q_{II}^{D} phase are indicated in blue.

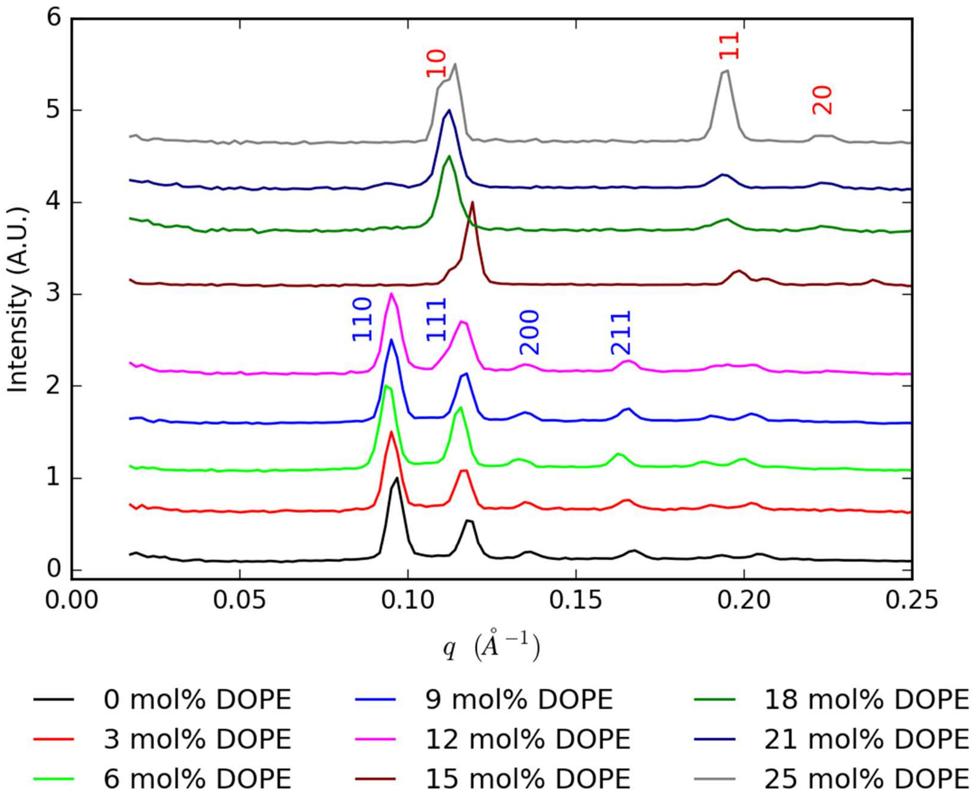

S5. 1D radially integrated scattering patterns of lipid systems of MO and DOPE as measured in water. Miller plane indices of the Q_{II}^{D} phase are indicated in blue, and Miller plane indices of the H_{II} phase are indicated in red.

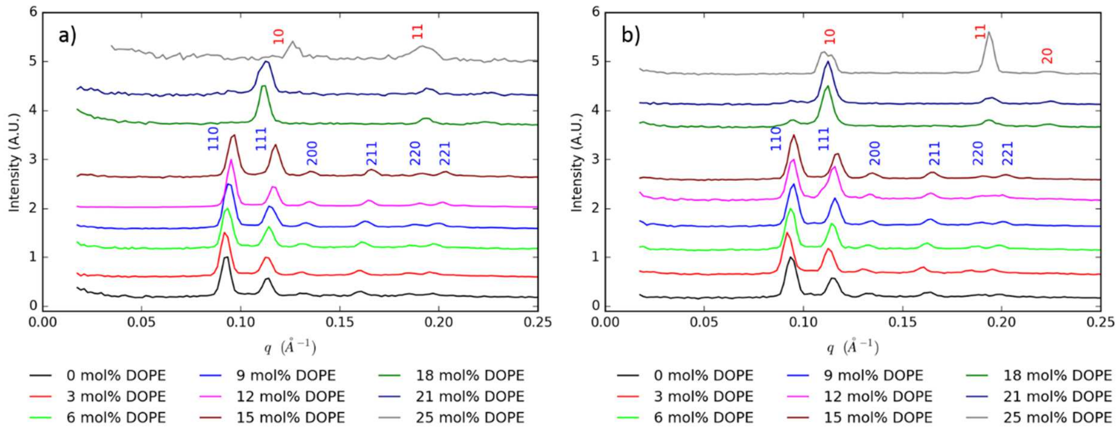

S6. 1D radially integrated scattering patterns of lipid systems of MO and DOPE in two concentrations of $CaCl_2$. (a) 0.01M $CaCl_2$; (b) 0.1M $CaCl_2$. Miller plane indices of the Q_{II}^{D} phase are indicated in blue, and Miller plane indices of the H_{II} phase are indicated in red.

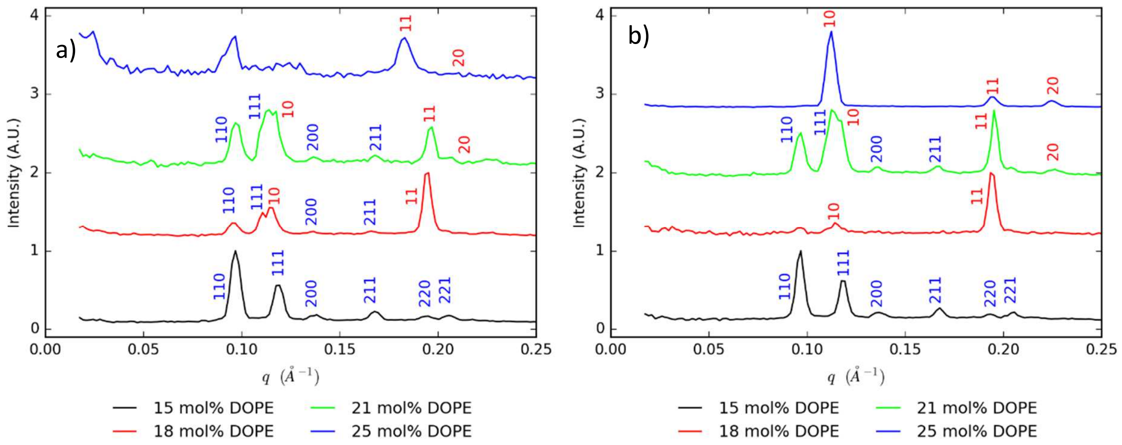

S7. 1D radially integrated scattering patterns of systems of MO and DOPE measured in several different proportions of DOPE around the phase boundary of the H_{II}-Q_{II}^{D} phase transition as measured in water, and two different concentrations of LiCl. (a) MO/DOPE systems in 0.01M LiCl; (b) MO/DOPE systems in 0.1M LiCl. Miller plane indices of the Q_{II}^{D} phase are indicated in blue, and Miller plane indices of the H_{II} phase are indicated in red. It should be noted that the first apparent peak in (a) at 25 mol% DOPE is a reflection from the beamstop.

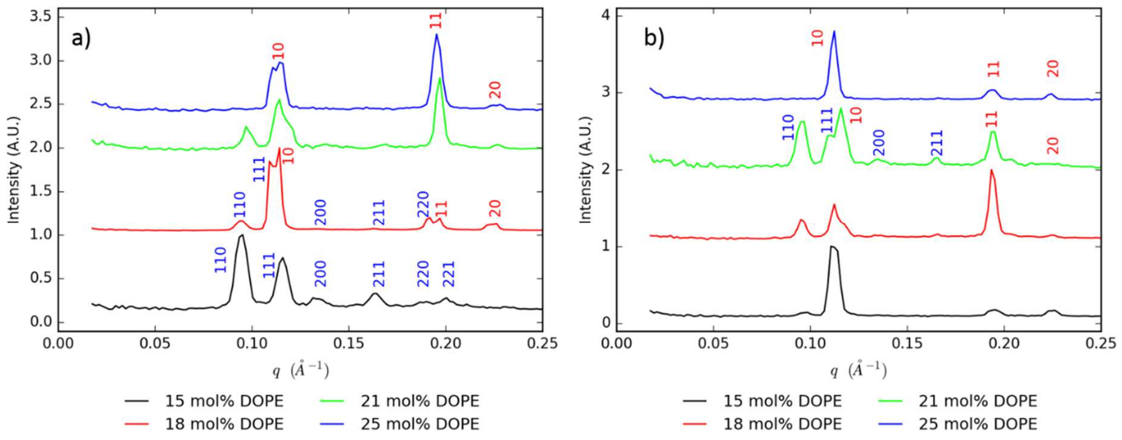

S7. 1D radially integrated scattering patterns of systems of MO and DOPE measured in several different proportions of DOPE around the phase boundary of the H_{II}-Q_{II}^{D} phase transition as measured in water, and two different concentrations of NaCl. (a) MO/DOPE systems in 0.01M NaCl; (b) MO/DOPE systems in 0.1M NaCl. Miller plane indices of the Q_{II}^{D} phase are indicated in blue, and Miller plane indices of the H_{II} phase are indicated in red.
